# Sperm Quality in Young Bull Semen Can Be Improved by Single Layer Centrifugation

**DOI:** 10.3390/ani12182435

**Published:** 2022-09-15

**Authors:** Isabel Lima-Verde, Emma Hurri, Theodoros Ntallaris, Anders Johannisson, Hans Stålhammar, Jane M. Morrell

**Affiliations:** 1 Clinical Sciences, Swedish University of Agricultural Sciences, Almas Allé 8, 750 07 Uppsala, Sweden; 2 VikingGenetics, 532 94 Skara, Sweden

**Keywords:** sperm quality, young bull ejaculates, genomic selection, genetic progress, generation interval, single-layer centrifugation, sperm cryopreservation

## Abstract

**Simple Summary:**

Genomic selection enables bulls with desirable genes to be identified early in life. Livestock producers need to use the semen from young bulls as early as possible for efficient milk and meat production with fewer greenhouse gas emissions. However, semen from young bulls is often of lower quality than needed for freezing for commercial artificial insemination. Colloid centrifugation selects spermatozoa with the desirable characteristics needed for fertilization from the rest of the ejaculate. In this study, split ejaculates from young bulls were prepared with or without colloid centrifugation. Using this technique, sperm doses of acceptable quality for artificial insemination could be produced from ejaculates that would otherwise be discarded. Thus, the semen from young bulls would be usable for artificial insemination sooner than is currently the case.

**Abstract:**

Interest in using semen from young bulls is increasing due to identifying promising animals by genomic selection. However, sperm quality in these ejaculates may not reach currently accepted standards for the cattle breeding industry. The purpose of this study was to determine if centrifugation of semen from young bulls through the Bovicoll colloid could improve sperm quality sufficiently for the frozen semen to be acceptable for artificial insemination. Ejaculates from 19 young bulls were split and either processed by Single-Layer Centrifugation (SLC) or not (CON) before freezing. After thawing, sperm quality was evaluated by determination of membrane integrity, mitochondrial membrane potential, DNA integrity, production of reactive oxygen species, sperm morphology and motility. Approximately half of the CON samples reached acceptable post-thaw quality (membrane integrity ≥ 40%) despite being below the breeding company´s desired sperm concentration threshold pre-freezing. In the remaining samples, sperm quality was improved by SLC such that 45% of them reached acceptable quality post-thaw. Almost 75% of the young bull sperm samples could have produced usable frozen semen doses by adjusting the breeding company´s current processing protocols. Since lowering the generation interval has a direct effect on the genetic gain per year, SLC could aid genetic progress in cattle breeding.

## 1. Introduction

Whereas artificial insemination (AI) with cryopreserved bull sperm revolutionized the cattle breeding industry in the 20th century [1], genomic selection is reputed to have achieved a similar effect early in the 21st century [2]. The genetics of both the cow and the bull are strongly implicated in the productivity of dairy cattle, i.e., the ability of the cow to conceive and maintain a pregnancy, and subsequently to become pregnant again while lactating. Moreover, genetics are involved in decreasing greenhouse gas emissions [3], since more efficient production can decrease the overall emissions per kg of meat or milk produced. Genetic selection for disease resistance and udder health could reduce antibiotic usage. Therefore, the choice of bull for a particular cow is not only of prime importance in reproductive efficiency but also in determining the effect of livestock production on the environment [4]. 

The advent of genomic selection enables future breeding sires to be identified at a young age [5]; there is considerable interest in being able to use these young bulls as semen donors for AI as soon as possible, to maximize the rate of genetic gain [6]. However, sperm quality is crucial in determining fertility after AI, although opinions differ about the sperm characteristics that are important in predicting fertilizing ability [7]. Unfortunately, the semen characteristics of young bulls tend to be inferior to older bulls, e.g., in sperm concentration and motility [8], as well as morphology [9]. As a result, such semen samples may not reach the breeding company´s quality control standards, either before freezing or after thawing [10]. 

Various methods are available to select spermatozoa with desirable characteristics from sperm samples [11]. A method of sperm processing, centrifugation through a single layer of colloid (Single Layer Centrifugation, SLC), was shown to separate robust spermatozoa from the rest of the ejaculate in a variety of species, including bovine bulls [12]. In this method, the extended semen sample is layered over a colloid and subjected to gentle centrifugation. Under centrifugal force, robust spermatozoa move through the colloid to form a pellet, while seminal plasma is retained on top of the colloid. Dead or dying spermatozoa are retained at the interface between the sample and the colloid, appearing as a white line [11]. 

In a previous experiment with semen from mature bulls, SLC was shown to select spermatozoa with better chromatin integrity (lower DNA fragmentation index) than the corresponding controls (uncentrifuged spermatozoa) and there was a higher proportion of spermatozoa with high mitochondrial membrane potential in SLC than the control samples [13]. Other sperm characteristics were not different between control and SLC samples. In another study, the selected SLC bull sperm samples were found to have a higher superoxide production and higher mitochondrial membrane potential than the controls, suggesting that the selected spermatozoa may have a higher metabolic activity than non-selected spermatozoa [14]. These studies led us to speculate that SLC might be useful in selecting good quality spermatozoa from the semen of young bulls that did not reach the breeding company´s thresholds for freezing. The company´s criterion for acceptance of the sample was a post-thaw membrane integrity (MI) of at least 40% [15].

Our hypothesis, therefore, was that SLC would enable ejaculates to be frozen that would otherwise be discarded. The purpose of this study was to determine whether sperm quality from young bulls could be improved by SLC prior to freezing, to the extent that the post-thaw quality would be acceptable for commercial semen doses for AI. 

## 2. Materials and Methods

### 2.1. Semen Collection

**Figure 1:**
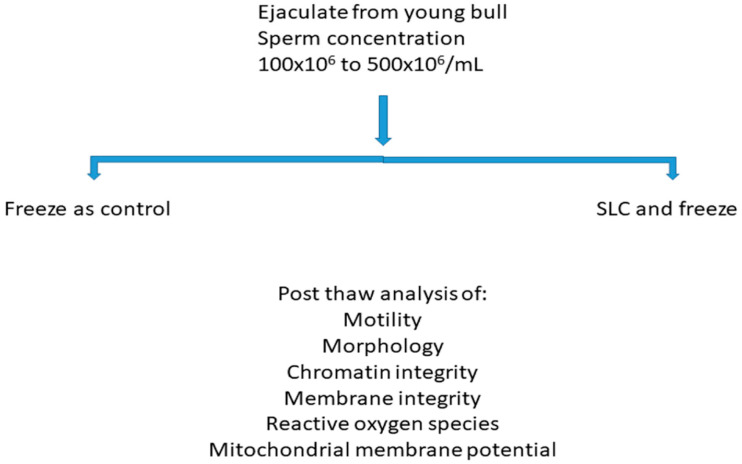
Experimental design. Note: SLC—Single layer centrifugation.

Nineteen young bulls at a commercial semen collection station were available for this study at the age of eight months. They were housed in a barn in individual pens and fed grass silage plus concentrates with a vitamin and mineral supplement. They were trained in the semen collection barn once a week by allowing them to mount teaser animals; once stimulated, they ejaculated into an artificial vagina. If an ejaculate was obtained, it was checked for the presence of spermatozoa; samples with a sperm concentration of more than 100 × 10^6^/mL were used for the present study. Samples with a sperm concentration of more than 500 × 10^6^/mL were not available for this study as they were required for freezing for commercial purposes. The experimental design is shown in Figure 1. Semen collected by an artificial vagina does not require ethical approval in Sweden.

### 2.2. Single-Layer Centrifugation

The sperm concentration was adjusted to 69 × 10^6^ spermatozoa/mL with Andromed™ extender (Minitűb International, Tiefenbach, Germany) before layering part of the sample over 4 mL Bovicoll colloid at a density of 1.088 g/mL. The remaining part of the sample served as the unselected control. After centrifugation at 300× *g* for 20 min, the supernatant was removed, and the sperm pellet was aspirated into fresh extender; the sperm concentration was adjusted to 69 × 10^6^ spermatozoa/mL. Both the control (uncentrifuged) and SLC samples were frozen in 0.25 mL plastic straws following the breeding company´s usual protocol and were stored in liquid nitrogen pending analysis. Note that the recommended density of colloid for 15 mL tubes for bull semen is 1.104 g/mL [12] but the first sperm preparations done with this density of colloid did not result in any spermatozoa passing through the colloid. Therefore, a colloid of density 1.088 g/mL was used in the present study, to allow more spermatozoa to pass through the colloid.

### 2.3. Sperm Analyses

The straws were transported to the Swedish University of Agricultural Sciences (SLU) in Uppsala. They were thawed at 37 °C for 12 s for the following sperm analyses. 

### 2.4. Sperm Concentration

Sperm concentration was measured using the Nucleocounter SP100 (Chemometec, Allerød, Denmark [13]. Briefly, 50 µL of semen were mixed with 0.5 mL detergent (Reagent S100; Chemometic). The sperm nuclei were stained by loading into cassettes containing propidium iodide, as supplied by Chemometic; the resulting fluorescence was measured using the fluorescence meter and the readout was converted to sperm concentration.

### 2.5. Flow Cytometry

The production of reactive oxygen species (ROS), sperm plasma membrane integrity (MI), mitochondrial membrane potential (MMP), and DNA fragmentation index (%DFI), were evaluated by flow cytometry after staining with fluorescent probes [13]. The sperm concentration was first adjusted to 2 × 10^6^/mL for MI and ROS, and to 2.5 × 10^6^/mL for MMP.

### 2.6. Production of Reactive Oxygen Species

Aliquots were stained with Hoechst 33258 at 1.2 μM (HO; Sigma, Stockholm), hydroethidine at 1.2 μM (HE; Invitrogen Molecular Probes, Eugene, OR, USA) and dichlorodihydrofluorescein diacetate at 60 μM (DCFDA; Invitrogen Molecular Probes) [16]. The samples were incubated at 37 °C for 30 min before analysis using a FACSVerse (BDBiosciences) flow cytometer (FC). Excitation was with a blue laser (488 nm) and a violet laser (405 nm). The detection of green fluorescence (FL1) was through a band-pass filter (527/32 nm); red fluorescence (FL3) was measured using another band-pass filter (700/54 nm); and blue fluorescence (FL5) was detected by a third band-pass filter (528/45 nm). In total, 30,000 events were evaluated. After gating for spermatozoa in the FSC-SCC dot plot, they were classified as live superoxide-negative, live superoxide-positive, dead superoxide-positive, live H_2_O_2_-negative, live H_2_O_2_-positive, dead H_2_O_2_-negative and dead H_2_O_2_-positive by establishing regions in the FL5/FL1 and FL5/FL3 dot plots. 

### 2.7. Membrane Integrity

Aliquots of all samples were stained with 0.08 µM SYBR 14 and 24 µM propidium iodide (PI; Live-Dead^®^ Sperm Viability Kit L-7011; Invitrogen, Eugene, OR, USA) [13]. After incubating in the dark at 37 °C for 10 min, the samples were evaluated using a FACSVerse flow cytometer (BDBiosciences; Franklin Lakes, NJ, USA). A blue laser (488 nm) was used for excitation. Green fluorescence (FL1) from SYBR 14 was detected with a 527/32 nm band-pass filter and red fluorescence (FL3) from PI was measured using a 700/54 nm band-pass filter. A total of 30,000 events were acquired for each sample. After gating to identify spermatozoa, the cells were classified as membrane intact (SYBR 14+/PI−), or membrane damaged (SYBR14−/PI+ or SYBR14+/PI+). In this study, only proportions of membrane intact spermatozoa are reported. 

### 2.8. Mitochondrial Membrane Potential

Aliquots of all samples were stained with the lipophilic cationic probe 5, 5′, 6, 6′-tetrachloro-1, 1′, 3, 3′-tetraethylbenzimidazolylcarbocyanine iodide (JC-1; Molecular Probes) at 12 µM [17]. After incubating the mixture at 37 °C for 30 min in the dark, analysis was carried out using a FACSVerse flow cytometer (BDBiosciences). Excitation of stained cells was obtained with a blue laser (488 nm); emitted fluorescence was detected using both FL1 (527/32 nm) and FL2 (586/42 nm) band-pass filters with compensation applied between channels. Following the evaluation of 30,000 cells, gating was performed to identify spermatozoa and to classify them into two groups: spermatozoa with high MMP (orange fluorescence) and those with low MMP (green fluorescence). Only the high MMP results are reported here.

### 2.9. Chromatin Integrity

The method is a modification of [18] as reported by [19]. Aliquots of sperm samples were mixed 1:1 with TNE buffer (Tris-sodium chloride-EDTA; 0.15 mol/L NaCl, 0.01 mol/L Tris-HCl, 1 mmol/L EDTA, pH 7.4), snap-frozen in liquid nitrogen and stored at −80 °C until analysis. After thawing the samples on ice, aliquots (10 μL) were extended with TNE buffer (90 μL) and subjected to partial DNA denaturation in situ with 0.2 mL of acid detergent solution (0.17% Triton X-100, 0.15 mol/L NaCl, and 0.08 mol/L HCl; pH 1.2). The samples were stained with acridine orange (0.6 mL; 6 μg/mL in 0.1 mol/L citric acid, 0.2 mol/L Na_2_HPO_4_, 1 mmol/L EDTA, 0.15 mol/L NaCl; pH 6.0). The samples were analysed using a flow cytometer (FACSVerse, BDBiosciences) within 3–5 min. For each sample, 10000 events were analysed at a speed of 200 cells/second using excitation with a blue laser (488 nm). The FSC (forward scatter), SSC (side scatter), FL1 (green fluorescence) and FL3 (red fluorescence) were collected. The DNA Fragmentation Index (%DFI—the ratio of cells with denatured, single-stranded DNA to total cells acquired, based on the ratio of red/red+green fluorescence for each cell), and the High DNA Stainability Index (HDS), were calculated for each sample using FCS Express version 5 (De Novo Software, Pasadena, CA, USA).

### 2.10. Morphology

Samples were available from 12 of the bulls for morphology. Smears were prepared from a drop of semen, air-dried and stained with carbolfuchsin-eosin [20]. Five hundred spermatozoa were evaluated under oil immersion at ×1000 magnification for morphological abnormalities such as proximal cytoplasmic droplets, detached heads, acrosome defects, nuclear vacuoles and tail defects. Spermatozoa were recorded as having normal morphology if no abnormality was detected. Further aliquots of semen were fixed in formol-saline and used to make wet mounts for evaluation of 200 spermatozoa at ×1000 magnification. The morphology evaluation was carried out by skilled personnel in the Swedish Sperm Reference Laboratory at SLU. 

### 2.11. Computer-Assisted Sperm Analysis

Sperm motility was analysed by computer-assisted sperm analysis (CASA) using a SpermVision analyser (Minitűb International, Tiefenbach, Germany) as described by Nongbua et al. [14]. An aliquot (5 µL) of each sample was placed on a warm glass slide on the heated stage (38 °C) of an Olympus BX 51 microscope. The kinematics of at least 1000 spermatozoa in eight fields were measured using the bull spermatozoa settings recommended by the manufacturer at a frame rate of 60/s. Particles with an area ranging from 20 to 100 μm^2^ were considered to be spermatozoa. Kinematics assessed were total motility (TM, %), progressive motility (PM, %) average path velocity (VAP; µm/s), curvilinear velocity (VCL; µm/s straight line velocity (VSL; µm/s), the ratios STR, LIN and WOB (VSL/VAP, VSL/VCL and VAP/VCL, respectively), amplitude of lateral head displacement (ALH; µm), and beat cross frequency (BCF; hz). Spermatozoa were identified as immotile (BCF < 0.2; VSL < 0.2), hypermotile (VCL > 80; LIN < 0.65; ALH > 6.5), and progressively motile (STR > 0.5 and LIN > 0.35).

### 2.12. Statistical Analysis

All statistical analyses were performed using SAS^®^ software (version 9.4; SAS Institute Inc., Cary, NC, USA). Descriptive statistics (mean, standard deviation, median) were calculated using the FREQ and SGPLOT procedure in the software. Data were analysed using PROC MIXED in the SAS software. The statistical model included the fixed effect of TIME (two classes; first or last ejaculate), TREATMENT (two classes; control or SLC) and their interaction. The model also included the random effect of the bull. The data that deviated from a normal distribution were log-transformed. However, to improve clarity, avoid redundancy and facilitate interpretation, the respective log-transformed values are referred and presented as untransformed values throughout the paper. Least-squares means (LSM ± SEM) estimated by the models were adjusted using the Scheffé adjustment for multiple post-ANOVA comparisons and compared. The alpha value for this experiment was selected to 5% and the p-values were compared based on the selected alpha value. Values for *p* ≤ 0.05 were considered significant. Differences for p between 0.05 and 0.10 were considered trends.

Pearson correlations were obtained using Excel software (Microsoft; Redmond, WA, USA).

Only the first and last ejaculates were analysed here because there were differences among bulls in the number of ejaculates collected. Since improvement in sperm quality was not linear, using the mean value of all ejaculates would not present an accurate picture of development with time.

## 3. Results

The number of ejaculates per bull varied, ranging from 1 to 10 per bull (Table 1). In total, 91 ejaculates were analysed.

### 3.1. Flow Cytometry

Sperm characteristics varied considerably among bulls and among ejaculates, tending to improve with the age of the bull (Table 2, and also Table 3, Table 4 and Table 5 in subsequent sections).

#### 3.1.1. Membrane Integrity, Chromatin Integrity, Mitochondrial Membrane Potential

Mean values for sperm membrane integrity in the controls ranged from 21% to 59% (Table 1). The treatment effect was not significant (*p* = 0.2), but the time of sampling and the time–treatment interaction were significant (*p* < 0.0001; *p* < 0.0049, respectively). Forty of the 90 control samples would not have reached the threshold of 40%. However, 18 of these 40 samples achieved ≥40% membrane integrity after SLC. Nine of the bulls (47%) achieved this value earlier in the SLC samples than in the controls, eight (42%) reached this value at the same time in the SLC and control samples, and two bulls (11%) reached this level later in the SLC samples than in the controls (Table 1).

The %DFI ranged differed markedly between the first and last ejaculates for both controls and SLC samples (*p* < 0.0006). The LSMean (±SEM) for %DFI for the first ejaculates was 14.13 ± 1.64 and 20.11 ± 1.64 for controls and SLC, respectively (*p* = 0.02), while for the last ejaculates the values were not different between treatments (9.79 ± 1.35 and 11.01 ± 1.35, respectively; *p* = 0.85). Similarly, for HDS the first ejaculates were significantly different between treatments (0.48 ± 0.04 and 0.77 ± 0.08 for controls and SLC, respectively; *p* < 0.0015), whereas the last ejaculates were not significantly different (0.48 ± 0.04 and 0.59 ± 0.06, respectively; *p* < 0.33). There was a trend towards significance for the time of sampling*treatment interaction (*p* = 0.0565).

Values for High MMP improved with the age of the bull, with no treatment effect or time of sampling*treatment interaction. For the first ejaculates, LSMeans (±SEM) were 26.2 ± 2.72% and 23.56 ± 2.45% for controls and SLC, respectively (*p* = 0.58), whereas for the last ejaculates LSMeans (±SEM) were 33.20 ± 2.8% and 34.07 ± 2.82%, respectively (*p* = 0.995).

**Figure 2:**
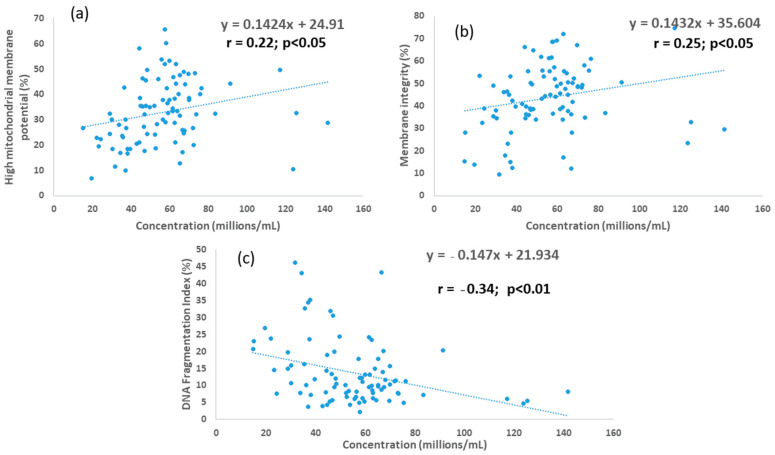
Associations between sperm concentration and (**a**) membrane integrity; (**b**) mitochondrial membrane potential; and (**c**) DNA fragmentation index for young bull ejaculates processed by single-layer centrifugation.

For SLC samples, sperm concentration was positively correlated with MI and high MMP (r = 0.22, r = 0.25, respectively; *p* < 0.05 for each) and negatively correlated with %DFI (r = −0.34; *p* < 0.01), as shown in Figure 2, whereas for the control, the association between sperm concentration and these parameters was not significant.

The improvement in chromatin integrity in SLC-selected samples and the proportion of spermatozoa with high MMP with age did not necessarily coincide with the improvement in MI. Bulls showing an improvement in SLC compared to control samples for all three parameters were bulls 1, 2, 10, 11 and 13 (Table 1), with similar sperm concentrations between control and SLC samples. Bulls showing a decrease in all three parameters in SLC samples compared to controls were bulls 3, 5, 15 and 17, with dissimilar sperm concentrations between the two sets of samples. Four bulls (7, 10, 14 and 16) showed an improvement in MI and MMP but not %DFI in the SLC samples compared to the controls, and three bulls (6, 9 and 12) had an improvement in %DFI in the SLC samples compared to controls but not in the other parameters. 

The improvement in chromatin integrity in SLC-selected samples and the proportion of spermatozoa with high MMP with age did not necessarily coincide with the improvement in MI. Bulls showing an improvement in SLC compared to control samples for all three parameters were bulls 1, 2, 10, 11 and 13 (Table 1), with similar sperm concentrations between control and SLC samples. Bulls showing a decrease in all three parameters in SLC samples compared to controls were bulls 3, 5, 15 and 17, with dissimilar sperm concentrations between the two sets of samples. Four bulls (7, 10, 14 and 16) showed an improvement in MI and MMP, but not %DFI in the SLC samples compared to the controls, and three bulls (6, 9 and 12) had an improvement in %DFI in the SLC samples compared to controls but not in the other parameters.

#### 3.1.2. Reactive Oxygen Species

All bulls showed a marked increase in the proportion of live superoxide-positive (live SO+) spermatozoa (*p* < 0.0001) and live hydrogen peroxide-negative spermatozoa (*p* < 0.03) in SLC samples compared to controls (Table 3). The time of sampling was significant (first and last ejaculates per bull for Control and SLC, *p* < 0.0005 and *p* < 0.0002, respectively) and there was a significant time of sampling*treatment interaction (*p* < 0.04). There was an association between sperm concentration and live H_2_O_2_- for both SLC and control samples, and also between sperm concentration and live SO+ for SLC samples.

### 3.2. Morphology

Ejaculates were analysed from each of the 12 bulls. Overall, the normal morphology (Table 4) was not different in the controls and SLC samples (60% vs 64%; *p* > 0.05) but differed between the first and last ejaculates (first: 56 ± 25% and 58 ± 26%; last: 70 ± 15% and 71 ± 17%, for the controls and SLC, respectively). The mean proportion of spermatozoa with proximal cytoplasmic droplets in the first ejaculates was 24.6 ± 12.3% in the controls and 21.2 ± 10.6% in SLC samples, decreasing to 12.9 ± 17% and 11.2 ± 15.8%, respectively, in the last ejaculates. Abnormal heads were 11.0 ± 7.1% versus 10.0 ± 5.0%, decreasing to 10.4 ± 10.9% and 6.3 ± 2.7%%, respectively, in the last ejaculates. The non-sperm cellular content (epithelial cells, spermatogenetic cells, etc.) was assessed qualitatively to be less in the SLC samples than the controls (first ejaculates with non-sperm cells 12/12 and 7/12 for controls and SLC, respectively; last ejaculates of 10/12 and 5/12 ejaculates, respectively).

### 3.3. Computer-Assisted Sperm Analysis (CASA)

**Figure 3:**
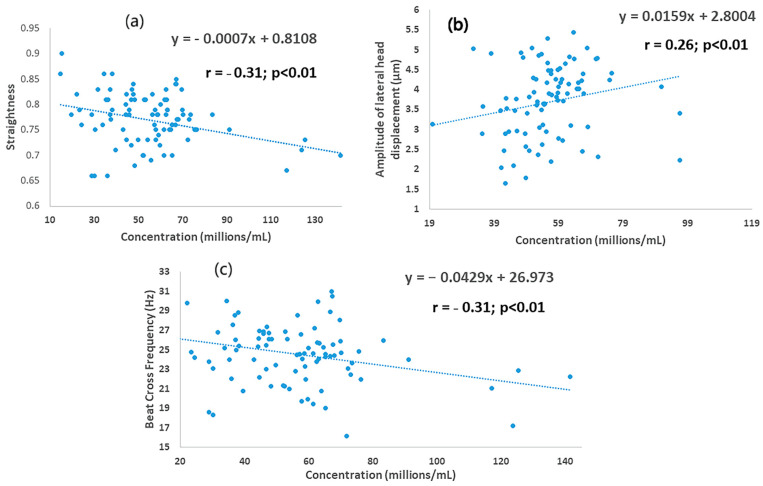
Association between sperm concentration and kinematics for young bull ejaculates processed by single-layer centrifugation: (**a**) straightness; (**b**) amplitude of lateral head displacement; (**c**) beat cross frequency.

The mean values for kinematics are shown in Table 5. The values for LIN and BCF were greater than in SLC samples than in the controls (*p* < 0.0001 for both parameters), whereas TM (*p* < 0.0048), PM (*p* < 0.0006) and WOB (*p* < 0.0252) were lower in SLC than the controls. The mean values for VAP, VCL, VSL, WOB and ALH were not different between treatments (*p* > 0.05). Most kinematics were affected by timing of sampling, i.e., values for first and last ejaculates, apart from VSL and STR; mean values were higher in the first ejaculate than for the last for LIN (*p* < 0.0057), WOB (*p* < 0.0019) and BCF *p* < 0.0024), whereas they were higher in the last ejaculate than the first for TM (*p* < 0.00001), PM (*p* < 0.0001), VAP (*p* < 0.0045), VCL (*p* < 0.008), and ALH (*p* < 0.0012). There was an interaction between timing of sampling and treatment for PM (*p* = 0.039) and LIN (*p* < 0.00064). Sperm concentration was correlated with STR, ALH and BCF in SLC samples (r = −0.30, r = 0.26, r = −0.31 respectively; *p* < 0.01 for each) but not in the controls (Figure 3).

## 4. Discussion

This study was performed to ascertain whether SLC could be used to improve the quality of young bull semen sufficiently for it to reach the standards required by commercial breeding companies to supply for AI. There was considerable variation regarding the effect of SLC, both among ejaculates and among bulls. As such, some parameters of sperm quality were improved in some ejaculates but not for other parameters. This result is in contrast to other studies with ejaculates from mature bulls where SLC produces a consistent improvement in sperm quality [14]. However, in the present study, there was a tendency for an improvement to be seen with age; thus, the later sample from each young bull was more likely to show an improvement after SLC than the earlier sample. The age at which this improvement occurred varied among bulls. However, there was also an association between sperm concentration and some sperm quality parameters for SLC samples but not for controls, which had a confounding effect on the comparison.

The major improvements after SLC were in the ROS status of the spermatozoa, with all bulls showing an increase in the proportion of live superoxide-producing spermatozoa and an increase in the proportion of live hydrogen peroxide-negative spermatozoa. This result is similar to the effect seen with fresh stallion spermatozoa [21] and is important for several reasons. The production of superoxide occurs as a metabolic byproduct: the higher sperm metabolism, the more superoxide is produced. Therefore, superoxide production can be considered to be an indicator of metabolism, with high levels indicating high metabolic activity. Previously, it was assumed that superoxide is immediately converted to hydrogen peroxide, which has a detrimental effect on sperm quality, damaging both sperm membranes and sperm chromatin. However, both in the previous study with stallion spermatozoa and in the present study with bull spermatozoa, there was no association between an increased superoxide production and hydrogen peroxide production, suggesting that superoxide may not be converted to hydrogen peroxide in spermatozoa. This finding is also in agreement with Macias Garcia et al. [22], who showed by fluorescence microscopy that superoxide and hydrogen peroxide are found in different compartments of the spermatozoon. There was an association between live superoxide-negative spermatozoa and live hydrogen peroxide-negative spermatozoa in the present study. It would have been interesting to investigate an association between the presence of non-sperm cells and production of hydrogen peroxide, but this was not possible in the present experiment due to the qualitative nature of the cellular assessment. 

There was a variable improvement in MI in the SLC-selected sperm samples, which may have been affected by the different sperm concentrations in SLC and control samples. For SLC samples, sperm concentration was positively correlated with MI and high MMP, and negatively correlated with %DFI, whereas there was no such association between sperm concentration and these parameters for controls. Therefore, the likely effects of sperm concentration on the sperm quality parameter being evaluated should be taken into account when interpreting the effect of SLC on sperm quality for these young bulls. Every effort was made to adjust the sperm concentration to 69 × 10^6^/mL in the pre-freezing samples, based on concentrations measured by flow cytometry at the bull station, but the post-thaw concentrations measured by the Nucleocounter showed that the sperm concentration in the post-thaw samples varied considerably. This variation may arise from an unequal distribution of spermatozoa in the sample bottle during straw filling, or from the different methods used to measure sperm concentration in the two laboratories. Although the association between concentration and various sperm quality parameters for SLC samples was significant, the correlation was weak. However, the take-home message is that the effect of concentration on SLC samples should be taken into account when comparing controls and SLC samples. 

Viability (or MI) is regarded as being a compensable defect [23], meaning that the fertility of samples with a lower than optimal MI can be boosted by increasing the number of spermatozoa in the AI dose. However, the indication that almost half of the control samples that did not reach the threshold for acceptance (≥40% MI) could be improved by SLC so that they exceeded the threshold for acceptance is very encouraging and could potentially be of interest to breeding companies. The use of SLC could mean that sperm samples could be used for commercial AI at the standard dose earlier than is currently the case.

In contrast to previous studies, there was no improvement in sperm morphology following SLC in this study. This finding could be attributable to the lower density colloid used in the present study than in previous studies; the lower density colloid was used in an effort to recover more spermatozoa in the pellet. However, lowering the density of the colloid tends to reduce its selective capacity, at least in the 15 mL tubes used in the present study. The density of the colloid needed to select robust spermatozoa depends partly on the size of tube used [24,25], although the size of the tube also affects sperm recovery [26]. 

There was an improvement in some kinematics following SLC in the present study, in line with previous studies. Thus, LIN and BCF were increased in the present study whereas VAP, STR, LIN, ALH and BCF were improved after SLC in a previous study using semen from mature bulls [27]. The total and progressive motility were lower in SLC samples in the present study, whereas they were unchanged in a previous study [13]. High values of LIN and BCF were previously associated with progressive motility [28], and would therefore be considered to be desirable, although the progressive motility in the present study was not increased compared to controls. This may be a reflection of the settings of the CASA instrument used. However, it is possible that the spermatozoa of young bulls are more vulnerable to the effects of centrifugation than the spermatozoa of older bulls, resulting in lower motility in some samples after SLC. Alternatively, the lower sperm concentration in some of the SLC samples might have introduced an artifact in this study, since the sperm motility would appear to be lower than in the controls. It might be necessary to adjust the composition of the extender to provide more protection to the spermatozoa during cryopreservation.

These results from SLC of young bull ejaculates are interesting for several reasons: despite the problem of not have matching concentrations in control and SLC samples, which potentially adversely affected the SLC results, it was possible to improve sperm quality by SLC sufficiently to obtain usable semen doses from some ejaculates. However, in other cases, it was also possible to obtain acceptable post-thaw quality from control samples that would have been discarded as not reaching the accepted arbitrary threshold of sperm concentration for freezing (500 × 10^6^/mL). This threshold is chosen by breeding companies to ensure that the amount of antibiotics and cryoprotectant in the extended sample is sufficient to protect the samples. If the sperm concentration is low, cryosurvival is impaired [29]. This observation implies that it should be possible to freeze some ejaculates from young bulls and obtain acceptable sperm quality, even without SLC, if the levels of cryoprotectant and antibiotics were adjusted according to the volume of the sample. Therefore, it could still be worth freezing the samples of low concentration, adjusting the proportion of cryoprotectant to compensate for the increased proportion of seminal plasma included, or alternatively, first removing the seminal plasma by gentle centrifugation through a colloid of lower density than the one used here. This procedure allows the majority of the spermatozoa to pass through the colloid while retaining the seminal plasma on top of the colloid [30]. The sperm pellet is then resuspended in cryomedium to give the desired sperm concentration without damaging the spermatozoa through sperm washing. The low-density colloid centrifugation technique has been used to separate stallion spermatozoa from cryomedium in a heterologous zona binding assay [31] and to separate bull spermatozoa from cryomedium for in vitro fertilization [32]. 

Overall, the results of this study show that with SLC treatment, semen from some young bulls can be approved for commercial use at a younger age than is currently possible. This is important for genetic progress per year as the age of the bull (generation interval) is the denominator in the equation for genetic gain. Half of the bulls in this study reached the threshold for acceptance of their thawed semen (≥40% membrane integrity) at a lower SLC age than the control age. In some cases, this difference in age is almost two months (54 days). Therefore, SLC has the potential to aid genetic progress in cattle breeding.

## 5. Conclusions

The effects of single-layer centrifugation were mixed, with positive responses for some parameters and no effect for other measures. In some cases, sperm motility was lower in SLC than the controls, but this may have been an artefact due to the lower sperm concentration. The ejaculates produced by young bulls varied considerably in sperm quality but, in some cases, this quality could be improved by SLC to reach the pre-freeze quality standards. Responses tended to be improved with increasing age at time of collection. However, since small numbers of spermatozoa are likely to be present, it might be necessary to hand-fill straws instead of using straw-filling machines. In this way, it would be possible for breeding companies to obtain small numbers of frozen sperm samples of acceptable quality using SLC when preparing sperm samples for freezing. This procedure could be introduced into the semen preparation laboratory without significantly disrupting the normal working routines. Lowering the generation interval will have a direct effect on the genetic gain per year as the generation interval is in the denominator in the breeder´s equation.

## Figures and Tables

**Table 1:** High mitochondrial membrane potential; membrane integrity and DNA fragmentation index (unadjusted means ± SD) for control and SLC-selected sperm samples (n = 91).

Bull	No. Ejacs	High MMP		Membrane Integrity		%DFI		Age* (days)	Age* (days)
		Control	SLC	Control	SLC	Control	SLC	Control	SLC
1	5	33 ± 9	36 ± 18	32 ± 13	44 ± 19	17.3 ± 10.8	14.4 ± 11.4	362	320
2	5	31 ± 4	39 ± 7	37 ± 12	48 ± 11	12.4 ± 4.1	10.3 ± 3.0	358	337
3	5	43 ± 15 ^a^	25 ± 3 ^a^	50 ± 17	37 ± 10	10.2 ± 3.6	14.4 ± 8.5	334	334
4	5	29 ± 13	27 ± 6	39 ± 11	44 ± 12	8.6 ± 2.4	12.4 ± 4.1	317	317
5	3	30 ± 17	20 ± 4	53 ± 3	40 ± 2	9.3 ± 2.0	10.4 ± 2.7	297	297
6	2	30 ± 8	24 ± 5	51 ± 7	44 ± 4	6.6 ± 1.2	5.8 ± 2.8	301	327
7	7	23 ± 9 ^a^	38 ± 12 ^a^	25 ± 13	40 ± 16	19.4 ± 4.0	25.6 ± 9.2	415	359
8	5	22 ± 5	25 ± 9	37 ± 14	36 ± 20	17.3 ± 8.0	22.9 ± 15.5	290	262
9	7	45 ± 5	39 ± 8	51 ± 5	47 ± 12	11.1 ± 1.4	10.4 ± 5.1	285	285
10	5	37 ± 13	39 ± 16	44 ± 15	51 ± 22	10.1 ± 2.6	10.9 ± 6.7	312	258
11	3	25 ± 7	39 ± 16	31 ± 15	46 ± 25	10.2 ± 2.1	8.0 ± 2.7	292	278
12	5	37 ± 7	31 ± 3	53 ± 4	46 ± 9	5.2 ± 0.9	5.0 ± 2.1	323	323
13	3	10 ± 10	21 ± 11	35 ± 11	36 ± 14	9.6 ± 4.2	8.2 ± 5.3	333	312
14	5	31 ± 7	37 ± 10	39 ± 7	48 ± 8	12.0 ± 2.1	15.7 ± 4.0	338	345
15	4	44 ± 4	34 ± 12	50 ± 7	42 ± 10	8.4 ± 1.9	12.8 ± 7.5	291	291
16	10	13 ± 8	27 ± 13	36 ± 11	42 ± 14	8.8 ± 2.0	11.7 ± 6.3	305	277
17	7	49 ± 11	47 ± 13	59 ± 15	56 ± 16	9.2 ± 5.8	9.4 ± 7.0	307	307
18	3	18 ± 5	17 ± 6	21 ± 11	24 ± 14	29.9 ± 4.4	36.6 ± 8.5	305	298
19	1	45	46	57	44	2.58	4.29	307	307

Note: ejac—ejaculate; SLC—single-layer centrifugation; MMP—mitochondrial membrane potential; MI—membrane integrity; %DFI—DNA fragmentation index. Age*—age of bull when membrane integrity was ≥40%, ^a^—signifcance.

**Table 2:** Membrane integrity, %DFI, high DNA staining and high mitochondrial membrane potential in first and last young bull ejaculates (n = 19 bulls).

Parameter	Timing of Collection	Control	SLC	P Treatment	P Timing	P Interaction
Membrane Integrity	First	34.84 ± 3.34	27.59 ± 2.64	0.0019	0.0001	0.0049
	Last	45.66 ± 2.79	47.68 ± 3.02	0.9406		
%DFI	First	14.13 ± 1.64	20.11 ± 1.64	0.0173	0.0006	0.0461
	Last	9.29 ± 1.35	11.01 ± 1.35	0.8461		
High DNA Staining	First	0.48 ± 0.04	0.77 ± 0.08	0.0015	0.1579	0.0565
	Last	0.48 ± 0.04	0.59 ± 0.06	0.33		
High MMP	First	26.2 ± 2.72	23.56 ± 2.45	0.58	0.00006	0.37
	Last	33.20 ± 2.82	34.07 ± 2.82	0.995		

Notes: SLC—single-layer centrifugation; MMP—mitochondrial membrane potential. Only the results for the first and last ejaculates from each bull were included in this analysis.

**Table 3:** Reactive oxygen species in SLC and control samples (LSMeans ± SE) in first and last young bull ejaculates (n = 19 bulls).

ROS Category	Timing	Control	SLC	P Treatment	P Timing	P Interaction
**Live SO−**	First	29.0 ± 2.2	16.2 ± 1.9	0.0004	0.0043	*p* = 0.03
	Last	33.4 ± 2.2	28.1 ± 2.1	0.25		
**Live SO+**	First	8.8 ± 1.2	23.6 ± 2.1	0.0001	0.0005	0.056
	Last	15.2 ± 1.1	26.2 ± 2.0	0.0001		
**Live H_2_O_2_^−^**	First	37.4 ± 3.0	38.4 ± 2.6	0.97	0.0002	*p* < 0.036
	Last	48.1 ± 2.2	54.5 ± 2.1	0.02		
**Live H_2_O_2_^+^**	First	0.08 ± 0.02	0.05 ± 0.02	0.55	0.52	*p* = 0.74
	Last	0.06 ± 0.02	0.03 ± 0.03	0.84		

Notes: ROS—reactive oxygen species; SO—superoxide; H_2_O_2—_hydrogen peroxide; SLC—single-layer centrifugation. Only the results for the first and last ejaculates from each bull were included in this analysis (Tining).

**Table 4:** Sperm morphology for control and SLC samples from young bull ejaculates (12 bulls).

	Timing	Control	SLC	P Treatment	P Timing	P Interaction
**Normal morphology (%)**	First	56 ± 28	58 ± 27	NS	*p* < 0.0001	*p* = 0.84
	Last	70 ± 16	71 ± 17	NS		
**Head defects (%)**	First	11 ± 7	10 ± 5	NS	*p* < 0.0001	*p* = 0.59
	Last	7 ± 3	6 ± 3	NS		
**Proximal cytoplasmic droplets (%)**	First	24 ± 25	21 ± 22	NS	*p* < 0.0017	*p* = 0.80
	Last	13 ± 18	11 ± 17	NS		

Note. SLC—single-layer centrifugation; first ejaculate—first time ejaculate had >100 × 10^6^ spermatozoa/mL; last ejaculate—ejaculate before reaching 500 × 10^6^ spermatozoa/mL. Only the results for the first and last bulls were included in this analysis.

**Table 5:** Sperm kinematics for control and single-layer centrifugation samples in first and last ejaculates (n = 91).

Parameter	Timing	Control	SLC	P Treatment	P Timing	P Interaction
TM (%)	First	30.8 ± 4.8	21.2 ± 3.2	*p* = 0.0048.	*p* < 0.00001	
	Last	50.8 ± 3.6	39.4 ± 3.9			
PM (%)	First	28.3 ± 3.4	16.1 ± 2.9	*p* = 0.0006.	*p* < 0.00001	*p* = 0.03
	Last	47.2 ± 3.4	33.8 ± 3.9			
VAP (µm/s)	First	49.4 ± 2.0	52.5 ± 2.4		*p* = 0.0045;	
	Last	59.0 ± 1.9	54.9 ± 2.1			
VCL (µm/s)	First	89.4 ± 4.4	97.0 ± 5.4		*p* = 0.008;	
	Last	110.0 ± 4.4	102.1 ± 4.8			
VSL (µm/s)	First	36.9 ± 1.6	42.4 ± 2.1			
	Last	42.4 ± 1.7	41.4 ± 1.8			
STR	First	0.74 ± 0.02	0.80 ± 0.01			
	Last	0.71 ± 0.01	0.74 ± 0.01			
LIN	First	0.41 ± 0.01	0.43 ± 0.01	*p* < 0.0001	*p* = 0.0057	*p* < 0.00064
	Last	0.38 ± 0.01	0.40 ± 0.01			
WOB	First	0.55 ± 0.01	0.54 ± 0.01	*p* < 0.0252	*p* = 0.0019	
	Last	0.54 ± 0.01	0.53 ± 0.01			
ALH (µm)	First	3.4 ± 0.2	3.5 ± 0.2		*p* = 0.0012	
	Last	4.4 ± 0.2	3.9 ± 0.2			
BCF (Hz)	First	23.3 ± 0.8	25.8 ± 0.9	*p* < 0.0001	*p* = 0.0024	
	Last	22.2 ± 0.6	23.7 ± 0.8			

Note. SLC—single-layer centrifugation; TM—total motility; PM—progressive motility; VAP—average path velocity; VCL—curvilinear velocity; VSL—straight line velocity; STR—straightness (VSL/VAP); LIN—linearity (VSL/VCL) and WOB—wobble (VAP/VCL); ALH—amplitude of lateral head displacement; BCF—beat cross frequency. Only the results for the first and last ejaculates are included in this analysis.

## Data Availability

The dataset supporting the conclusions of this article is included within the article.
